# The Anticancer Activities of Some Nitrogen Donor Ligands Containing bis-Pyrazole, Bipyridine, and Phenanthroline Moiety Using Docking Methods

**DOI:** 10.1155/2018/5796287

**Published:** 2018-06-04

**Authors:** Adebayo A. Adeniyi, Peter A. Ajibade

**Affiliations:** School of Chemistry and Physics, University of KwaZulu-Natal, Private Bag X01, Scottsville, Pietermaritzburg 3201, South Africa

## Abstract

The anticancer study of nitrogen-chelating ligands can be of tremendous help in choosing ligands for the anticancer metal complexes design especially with ruthenium(II). The inhibitory anticancer activities of some nitrogen-chelating ligands containing bis-pyrazole, bipyridine, and phenanthroline were studied using experimental screening against cancer cell and theoretical docking methods. *In vitro* anticancer activities showed compound **11** as the most promising inhibitor, and the computational docking further indicates its strong inhibitory activities towards some cancer-related receptors. Among the twenty-one modelled ligands, pyrazole-based compounds **7**, **11**, and **15** are the most promising inhibitors against the selected receptors followed by **18** and **21** which are derivatives of pyridine and phenanthroline, respectively. The presence of the carboxylic unit in the top five ligands that displayed stronger inhibitory activities against the selected receptors is an indication that the formation of noncovalent interactions such as hydrogen bonding and a strong electron-withdrawing group in these compounds are very important for their receptor interactions. The thermodynamic properties, the polarizabilities, and the LUMO energy of the compounds are in the same patterns as the observed inhibitory activities.

## 1. Introduction

**Figure 1:**
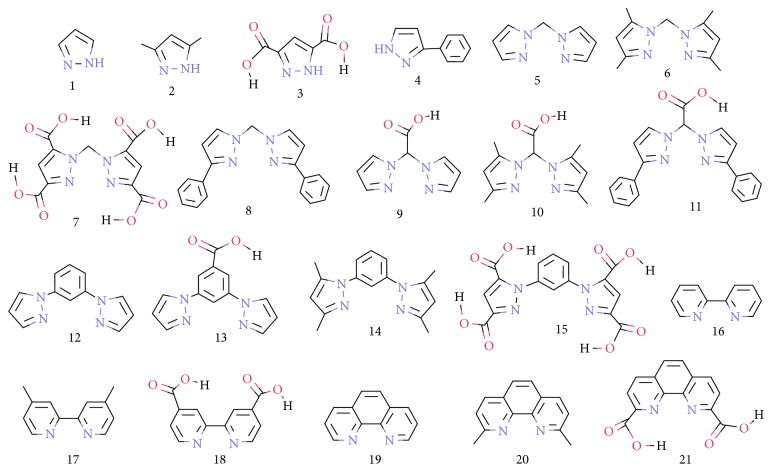
The schematic representation of the studied nitrogen-chelating ligands.

Nitrogen-chelating ligands such as bis-pyrazole (pz), bipyridine (bpyr), and phenanthroline (phn) derivatives are being used in the design of several metal complexes for ranges of applications from biological to nanostructured materials, especially the development of anticancer complexes of ruthenium [1–19]. Other studies have shown that these nitrogen-chelating compounds play significant roles in the experimentally observed anticancer activities [20] that might be ascribed to the electronic interactions between the metal centre and the *π*-electrons in rings [21–23]. However, despite the use of these nitrogen-chelating ligands in the design of anticancer metal complexes, there has been very few or no attention on the anticancer activities of these ligands individually. To this end, we have selected some derivatives of common nitrogen-chelating ligands as shown in Figure 1 to study their individual anticancer activities with particular focus on their interactions with cancer-related receptors using various docking methods. We also report the experimental *in vitro* anticancer activities of some of these ligands.

The selected receptors used for the docking studies are carbonic anhydrase II (CA-II), cathepsins B (Cat B) [24], two different DNAs (DNA-1 [25] and DNA-2), DNA gyrase (Gyrase) [26], histone deacetylase7 (HDAC7) [27], histone protein in the nucleosome core particle (HIS) [28], BRAF kinase (Kinase) [29], recombinant human albumin (rHA) [30], ribonucleotide reductase (RNR) [31], topoisomerase II (Top II) [32], thioredoxin reductase (TrxR) [33], and thymidylate synthase (TS) [34]. These receptors play significant roles in cancer growth and are thus a unique target in cancer therapy. For instance, rHA plays a significant role in the pharmacokinetic availability, bioavailability, and toxicology [35] and helps either in delivery of metal-based anticancer drugs to their cellular targets or in deactivating them even before reaching the target(s) [36]. The RNR is responsible for the synthesis of DNA from the corresponding building blocks of RNA [37]. Top II plays a key role in relaxing supercoiled DNA for replication and transcription in the absence of inhibitors [38], while the presence of inhibitors forms a stable complex with the enzyme and keeps it from DNA cleavage [39]. Thioredoxin reductase (TrxR) regulates the cellular reduction/oxidation (redox) status [40, 41]. Thymidylate synthase (TS) is a critical enzyme in maintaining a balanced supply of deoxynucleotides required for DNA synthesis and repair [42]. It is a target of chemotherapy to test the vulnerability of cancer cells to the inhibition of TMP synthesis [37]. Gyrase was considered to establish possible dual roles for the ligands as potential antibacterial agents.

## 2. Synthesis and Structural Elucidation of the Ligands

The synthesis and careful structural elucidation of these ligands have been reported in our previous works [43–45].

## 3. Experimental Methods

The *in vitro* anticancer activities of the ligands against the cancer cell line HT29 and normal cell line KMST were examined using the MTT colorimetric assay. All the ligands before their docking to the receptors were first optimized with DFT functional PBEPBE [46] and the basis set 6-31 + G(d,p) for all atoms using the Gaussian 09 package [47]. The docking analyses were carried out using Molegro [48] and Vina and AutoDock [49] packages. The docking of each ligand against the receptors was done five times: twice in Molegro first using the quantum Mulliken atomic charges for all atoms of each ligand (subsequently referred to as Molegro-QC) and second using the predicted atomic charges from the Molegro package (referred to as Molegro). One time in the Vina package using the predicted atomic charges from AutoDock tools and twice in AutoDock using both the QM charges (AutoDock-QC) and package-predicted atomic charges (AutoDock) has done in Molegro.

The default parameters were used in the Vina docking package but with little modifications in Molegro and AutoDock dockings. The scoring function used in Molegro was MolDock because it takes care of the hydrogen bonding, intermolecular protein ligand, and intramolecular ligand interactions and has been successfully applying for molecular docking [50]. The maximum interaction was set to 2500 instead of the default value of 1500, and the population number was increased from the default value of 50 to 100. In using AutoDock, the number of grid points in *x-*, *y-*, and *z*-axes was set to 60 × 60 × 60 with each point separated by 0.375 Å. The Lamarckian genetic algorithm was chosen based on its efficiency and reliability in comparison with others like simulated annealing (SA) and generic genetic algorithm (GA) methods in AutoDock [51, 52]. The maximum number of energy evaluations was set to 2,500,000 for each of the 20 independent runs, a maximum number of 27,000 GA operations were generated on a single population of 100 individuals, and step sizes of 2 Å for translation and 50° for rotation were chosen.

All the graphical representations of the docking results are prepared using the package Chimera [53].

## 4. Results and Discussion

Twenty-one ligands were modelled which comprise fifteen models of bis-pyrazole, three models of bipyridine (bpyr), and three models of phenanthroline (phn) (Figure 1). Six of the modelled compounds were screened to determine their *in vitro* anticancer activities, and their results are presented in Table 1. The *in vitro* activities of these ligands were tested against the cancer cell line HT29 and the normal cell line KMST using the MTT colorimetric assay. The results clearly showed that the six ligands pose no threat to the normal cell line as their inhibitory activities (IC_50_) in KMST are found to be greater than 50 *μ*m as shown in Table 1. Ligands **11** and **14** are the most potent (<6.25 mm) against the cancer cell line HT29, while ligand **12** is the least active. Also, the same ligand **11** was found among the three best inhibitors of the selected receptors from the docking results (Table 2).

### 4.1. The Binding Site Predictions of the Compound from the Docking Methods

**Figure 2:**
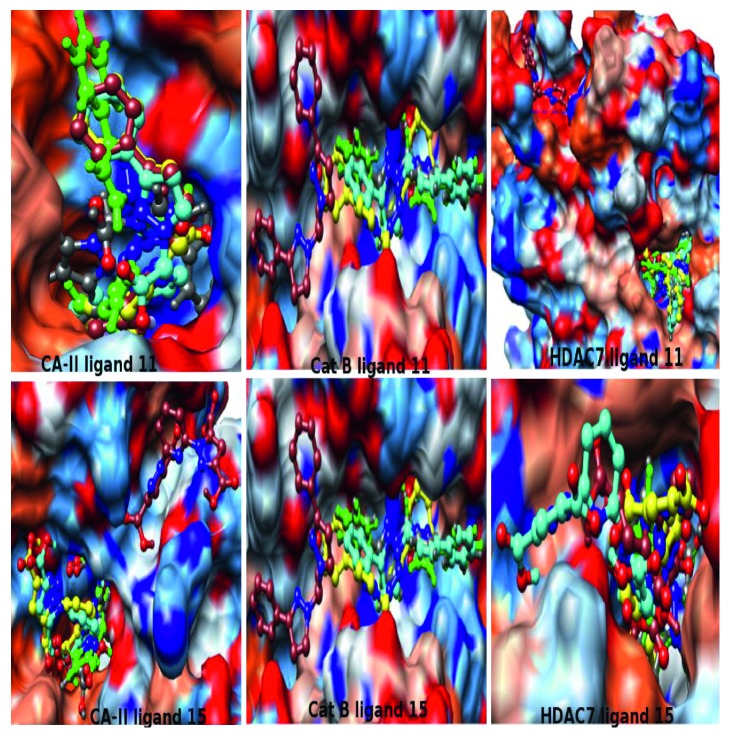
The binding site interaction of the ligands **11** and **15** with CA-II, ligand **11** with Cat B, and ligands **11** and **15** with HDAC7 using Molegro-QC (grey), Molegro (green), Vina (brown), AutoDock-QC (yellow), and AutoDock (cyan) methods.

The results obtained from the different docking methods and packages: Molegro-QC (grey), Molegro (green), Vina (brown), AutoDock-QC (yellow), and AutoDock (cyan), are shown in Figure 2. The features of the interactions of the ligands with the receptors using different docking methods are similar. In most of the receptors, the same binding sites were located by docking methods, and very similar conformational orientations of the ligands in the binding sites were predicted with some found to overlap each other (Figure 2). Ligands **11** and **15** have similar binding orientation in CA-II, Cat B, and HDAC7 based on the results obtained from the binding site interactions. Compound **11** is of interest because it shows promising anticancer activities *in vitro*, and ligand **15** is predicted as the best inhibitor of many of the receptors according to the results obtained from Molegro-QC and Molegro. The interaction of **11** with CA-II shows that all the docking methods gave similar orientation for the compounds except Molegro-QC (grey) that is slightly different in orientation (Figure 2).

The orientation obtained from Molegro-QC (grey) gives the best interacting energy compared to others (Table 2). Also in the interaction of **15** with CA-II, all of the methods locate the same binding site and give similar orientation of the ligand except Vina (brown) which locates a different binding site compared to others. The Molegro-QC and Molegro orientations of **15** are superimposed and likewise AutoDock and AutoDock-QC. All the five methods except Vina also locate the same binding site for the interaction of **11** with Cat B, and AutoDock prediction was found to be superimposed with that of Molegro-QC and Molegro. The results obtained from AutoDock predict different binding sites for the interaction of **15** with Cat B, while all other methods predict the same site for its binding. Another receptor of interest is HDAC7 in which strong inhibitory activities are displayed by **11** and **15**. HDAC7 was predicted as one of the most targeted receptors for many of the ligands according to the results obtained from Molegro-QC and Molegro. Vina locates a completely different binding site for the interaction of **11** with HDAC7 when compared to the rest of the methods. The binding site orientation of **11** from all the methods besides Vina is very similar to that of Molegro-QC completely superimposed with that of AutoDock. All methods predicted a similar binding site orientation of the ligand **15**, of which the Molegro-QC and Molegro orientations are found to be superimposed and in close orientation with those of Vina and AutoDock-QC, which are different from those of AutoDock. There is a significant difference between the results from AutoDock-QC and AutoDock methods in terms of the features of the ligand interactions with receptors just because of changes in the accuracy of the atomic charges. The correlation of the results of AutoDock-QC and AutoDock ranges from −0.32 to 0.46, while that of Molegro-QC and Molegro ranges from 0.96 to 1.00. It is obvious that the accuracy of atomic charges plays a very strong role in the determination of the ligand interaction with the receptors especially when using AutoDock. The results show that the reason for the inconsistency from Vina, AutoDock-DC, and AutoDock could be ascribed to different binding sites which were predicted for many of the ligand interactions with the receptors, while high consistency and similarity were obtained in Molegro-QC and Molegro because they both predicted the same binding site as cocrystallized inhibitors of the receptors. In addition, the differences in the ligand-receptor inhibitory energies obtained from AutoDock methods for many of the ligands are within the standard error margin of ∼2.177 kcal/mol [43, 44] that makes the order obtained from AutoDock unreliable.

### 4.2. The Inhibitory Activities

**Figure 3:**
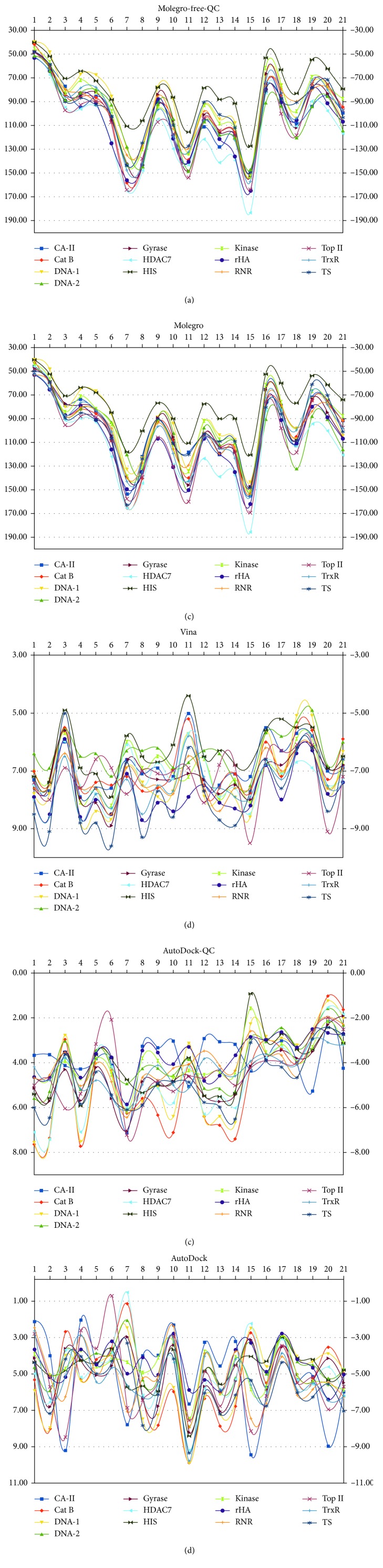
The plots of the energy of interaction of the ligands with each of the receptors using the five methods.

The ligands **7**, **11**, and **15** have the best inhibitory activities towards many of the receptors according to the results obtained from Molegro-QC and Molegro (Table 2 and Figure 3). Besides the first three bis-pyrazole ligands, next promising ligands in interaction with the selected receptors are **18** and **21** which are bpyr and phn compounds. The common feature possessed by all the promising ligands is carboxylic acid moieties which are predicted to enhance the noncovalent interactions such as hydrogen bonding and play the role of electron withdrawing. Since the results from the two methods Molegro-QC and Molegro are highly correlated (0.96 to 1.00), Molegro-QC is then used for the analysis of the receptor interaction of ligands **7**, **11**, and **15**. Although in many of the ligand interactions with the receptors, AutoDock and Vina gave similar features in their interaction, the ranking of the ligands according to their inhibitory interaction is vague. It has been pointed out that, to have a good ranking in AutoDock, the error values of ∼2.177 kcal/mol [43, 44] have to be considered, and this makes AutoDock unsuitable for ranking of these ligands because the differences in their interacting energy are lower.

**Figure 4:**
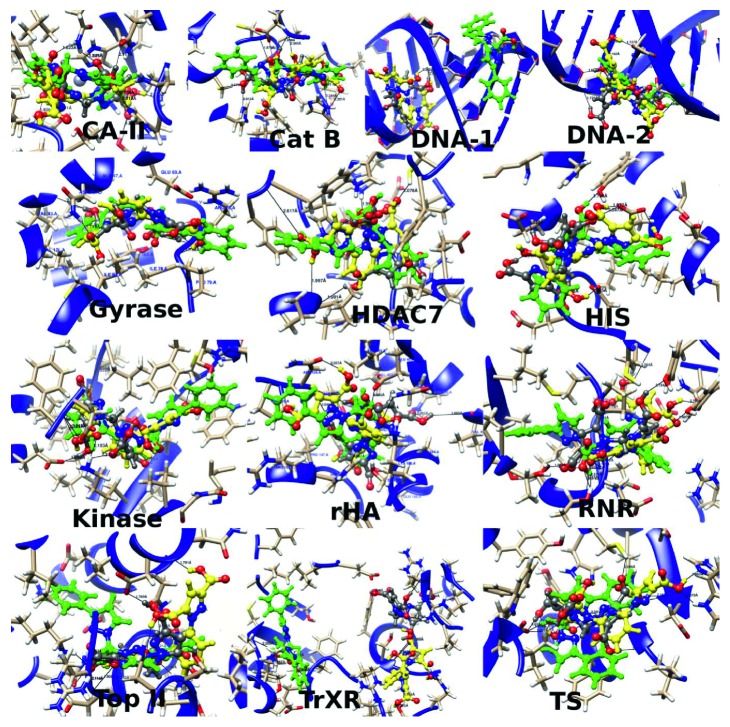
The binding site interactions of the three ligands **7** (grey), **11** (green), and **15** (yellow) with each of the receptors.

The three best inhibitors among all the modelled compounds are the bis-pyrazole-based compounds **7**, **11**, and **15** (Figure 3). The binding site interactions of these three ligands with each of the receptors are shown in Figure 4, while the interactions of each of the compounds **7**, **11**, and **15** with each of the receptors are shown in supplementary Figure S1. In most of the receptors, the three compounds **7** (grey), **11** (green), and **15** (yellow) are found to be clustering around the same point and sharing common angles of interaction with the receptors CA-II, Cat B, DNA-2, Gyrase, HDAC7, HIS, Kinase, rHA, RNR, Top II, and TS (Figure 4). The few exceptions to these common interaction features are found in the receptors DNA-1 and TrxR (Figure 4). A different binding site was predicted for the interaction of **11** with DNA-1 where **7** and **15** have inner groove interaction but the interaction of **11** is out of groove. In addition, the binding site predicted for **11** is separated from the one predicted for **7** and **15** in the receptor TrxR. In all of the receptor interactions especially for **7** and **15**, the predicted binding sites are the same as the binding site for their cocrystallized inhibitors except in the receptor DNA-2 where the three ligands bind to a groove outside of the binding surface of the cocrystallized *cis*-platin.

The receptor interactions of the three most promising compounds **7**, **11**, and **15** follow a different order based on their receptor preferences. The order of their inhibitory activities in HDAC7, Kinase, rHA, RNR, TrxR, and TS is **15** > **7** > **11**, while they follow the order of **7** > **5** > **11** in their interaction with CA-II, Cat B, and Top. The interaction of **11** was found to be better than that of **7** with the DNA-1, Gyrase, and HIS in the order of **15** > **11** > **7**, and also, the inhibitory activity of **11** against DNA-2 is found to be higher than that of **7** and **15** in the order of **11** > **15** > **7**. The presence of more than one carboxylic unit in **7** and **15** compared to **11** results in a greater number of HB interactions (Table 3), which could also be responsible for their greater inhibitory activities compared to **11** in many of the receptors. The number of the HB interactions of the ligand **11** in most of the receptors ranges from 0 to 2 except in the receptors Top II, TrxR, and TS where their binding site residues support more hydrogen bond interactions with the ligands (Table 3 and Figure S1).

### 4.3. Molecular Properties of the Compound

The molecular properties of the modelled compounds are shown in Table 4. Compounds **13** and **14** have the highest hyperpolarizability values, while **13** and **21** have the lowest band gap, but **14** and **21** have the highest dipole. The hyperpolarizability of the ligands follows the order of **13** > **14** > **12** > **21**. These four ligands can be useful as building blocks for nonlinear optical materials, but only the ligand **21** appears among the best five inhibitors obtained from the docking results. In order to study the possible effects of changes in the molecular properties on the docking interactions with the receptors, the molecular properties of the compound correlated with the docking results obtained from the Molegro-QC method.

All the thermodynamic energy, zero energy, thermal energy, enthalpy, and the Gibbs free energy of the compound have a correlation range of 0.92 to 0.98 with the docking results. In addition, the CV and entropy highly correlate with the inhibitory values of the compounds in the ranges −0.90 to −0.96 for CV and −0.92 to −0.97 for entropy. Besides the thermodynamic properties, other properties that show high correlation with receptors' inhibitory activities are their polarizabilities (range from 0.74 to 0.90) and polarizabilities W (range from 0.78 to 0.93). Other computed properties like LUMO (0.46 to 0.64), gap (0.32 to 0.60), Hyp (−0.20 to −0.40), and dipole (−0.22 to −0.37) have very low correlation (as shown in the parenthesis). The HOMO has the poorest correlation with the binding activities of the ligands which range from 0.00 to 0.18. The better correlation of the LUMO with the receptor interactions further supports the hypothesis that the lower the LUMO energy of inhibitors the easier the overlapping of it with the HOMO of the DNA [54]. However, other factors like the hydrophobicity and electronic effect of the ligands play significant roles in their inhibitory activities [54].

## 5. Conclusion

The inhibitory potentials of nitrogen donor ligands in their interaction with twelve cancer-related receptors were studied using docking methods. Twenty-one derivatives of nitrogen-chelating ligands consisting of fifteen pyrazole, three bipyridine, and three phenanthroline derivatives were modelled. In addition, the experimental *in vitro* anticancer activities of the six ligands **5**, **6**, **9**, **11**, **12**, and **14** against the cancer cell line HT29 are discussed. The result of the *in vitro* study shows that the ligands **11** and **14** are the most active ones compared to other ligands. Among the two, only the ligand **11** showed promising inhibitory activities towards the selected receptors. In many of the receptors, different docking methods locate similar binding sites and similar ligand orientations. The order of the ligand interactions with the receptors using Vina, AutoDock-DC, and AutoDock is inconsistent because they locate different binding sites in many cases and their energy difference is also within the range of the standard error of AutoDock (∼2.177 kcal/mol), while the order from Molegro-QC and Molegro is highly consistent and similar.

The results from the Molegro-QC and Molegro showed that **7**, **11**, and **15** have better inhibitory activities towards many of the receptors and have similar angles of interaction with CA-II, Cat B, DNA-2, Gyrase, HDAC7, HIS, Kinase, rHA, RNR, Top II, and TS. This is an evidence that they have common residue interactions at the binding sites of each of the receptors. The receptor interactions of the three most promising ligands **7**, **11**, and **15** follow a different order based on their receptor preference. Apart from these three, **18** and **21** with bipyridine and phenanthroline moieties also show promising interaction with many of the receptors. The most common feature of the best five inhibitors is the carboxylic unit, which indicates that the carboxylic units enhance the binding site interaction of the ligands through formation of stronger HB interactions with the receptor residues. The thermodynamic properties have a high correlation with the docking results of the ligands' inhibitory activities. Other molecular properties, which have a high correlation with the ligands' inhibitory activities are polarizabilities. The LUMO of the ligands shows a good correlation with their receptor-binding interaction which further supports the hypothesis that the lower the LUMO energy of inhibitors the easier the overlapping of it with the HOMO of the DNA.

## Figures and Tables

**Table 1:** The experimental anticancer activities of selected ligands.

Name	Compound	IC_50_-KMST	IC_50_-HT29
5	bpzm	>50	6.68
6	bdmpzm	>50	7.12
9	bpza	>50	6.25
11	bphpza	>50	<6.25
12	bpzpy	>50	15.87
14	bdmpzpy	>50	<6.25

**Table 2:** The interacting free energy of the ligands with the receptors using the five docking methods approached.

Ligands	CA-II	Cat B	DNA-1	DNA-2	Gyrase	HDAC7	HIS	Kinase	rHA	RNR	Top II	TrxR	TS
*Molegro-QC*													
1	−46.28	−41.89	−40.28	−46.26	−47.44	−50.56	−40.36	−45.16	−53.17	−50.49	−48.58	−51.94	−49.68
2	−60.37	−60.30	−48.43	−58.25	−59.81	−67.45	−52.16	−56.96	−64.71	−60.84	−64.03	−65.38	−59.07
3	−77.18	−85.66	−79.92	−88.62	−83.09	−94.83	−70.38	−83.19	−89.26	−79.78	−97.24	−86.21	−89.74
4	−94.47	−86.37	−67.14	−82.41	−85.22	−95.87	−64.14	−71.90	−82.84	−82.26	−92.74	−78.40	−86.99
5	−85.49	−86.16	−67.78	−79.76	−82.00	−94.12	−72.68	−81.62	−90.88	−90.19	−89.03	−78.29	−92.50
6	−105.08	−107.91	−85.51	−92.88	−104.83	−123.77	−88.68	−97.99	−124.96	−103.38	−107.50	−101.76	−102.61
7	−156.65	−158.15	−130.71	−127.71	−141.14	−165.05	−110.63	−143.43	−155.81	−134.63	−163.91	−147.53	−143.67
8	−142.47	−144.76	−129.84	−144.30	−146.90	−147.75	−105.85	−130.09	−142.98	−127.19	−137.97	−141.72	−124.60
9	−93.35	−93.62	−78.27	−92.25	−83.92	−104.76	−78.17	−96.30	−87.97	−85.27	−107.07	−96.84	−90.63
10	−113.53	−109.57	−88.24	−104.42	−108.46	−129.40	−86.09	−112.86	−121.09	−108.90	−117.22	−119.46	−109.56
11	−142.68	−138.71	−135.09	−148.33	−148.01	−142.87	−115.42	−133.47	−140.45	−128.89	−153.61	−132.24	−126.99
12	−111.01	−104.12	−92.91	−106.53	−101.00	−121.63	−78.65	−92.82	−105.68	−97.98	−104.24	−105.62	−90.49
13	−127.70	−113.80	−104.98	−117.41	−115.64	−141.14	−87.77	−108.12	−121.31	−114.24	−114.03	−118.08	−100.97
14	−116.59	−116.18	−108.02	−120.02	−119.72	−139.34	−91.47	−113.85	−136.08	−115.29	−118.61	−122.42	−111.92
15	−151.38	−148.61	−154.18	−146.92	−153.19	−183.25	−127.38	−152.66	−164.79	−150.54	−163.67	−157.02	−150.51
16	−67.83	−79.06	−70.27	−90.64	−66.12	−82.86	−52.94	−61.67	−76.02	−77.82	−80.37	−72.42	−81.01
17	−85.51	−85.09	−76.06	−93.14	−82.76	−91.71	−63.40	−76.46	−90.42	−79.56	−99.96	−82.29	−87.81
18	−108.41	−104.64	−104.01	−119.95	−112.01	−110.74	−83.28	−98.06	−105.46	−89.43	−118.24	−104.13	−90.52
19	−94.06	−74.75	−77.07	−93.66	−79.56	−89.73	−54.71	−69.36	−78.56	−79.77	−75.01	−71.60	−78.72
20	−85.01	−77.39	−78.52	−85.22	−82.85	−97.48	−62.38	−76.30	−91.29	−79.35	−75.30	−79.85	−71.57
21	−99.68	−94.52	−107.46	−114.19	−108.07	−116.87	−79.39	−87.55	−106.93	−102.49	−97.32	−97.06	−103.61

*Molegro*													
1	−45.66	−42.21	−40.15	−46.30	−47.06	−49.96	−40.40	−43.52	−52.79	−53.06	−48.11	−52.08	−49.85
2	−59.33	−61.35	−48.45	−57.94	−59.04	−66.37	−52.56	−56.81	−65.42	−60.80	−61.96	−65.28	−59.28
3	−78.69	−86.29	−80.14	−87.82	−76.80	−91.25	−70.80	−84.05	−87.22	−88.79	−95.38	−87.92	−89.71
4	−73.50	−82.40	−65.33	−81.76	−78.29	−89.64	−63.70	−71.11	−80.07	−80.36	−88.19	−76.57	−85.37
5	−84.53	−86.07	−67.14	−81.79	−81.35	−93.52	−68.36	−82.27	−91.04	−83.31	−88.91	−82.65	−91.05
6	−104.61	−108.91	−86.76	−93.07	−103.26	−122.10	−85.12	−96.98	−116.04	−99.66	−108.78	−101.37	−107.32
7	−153.41	−163.83	−133.00	−120.14	−139.49	−165.52	−118.00	−139.06	−149.63	−140.05	−157.74	−142.84	−162.70
8	−122.26	−141.64	−123.08	−138.05	−137.15	−144.97	−100.60	−131.59	−134.89	−127.66	−139.28	−135.21	−123.90
9	−92.83	−93.73	−88.77	−89.42	−89.03	−106.92	−76.99	−93.14	−107.05	−94.00	−106.00	−96.60	−90.55
10	−113.37	−109.72	−94.83	−102.39	−105.85	−129.22	−90.12	−115.29	−131.03	−116.72	−130.17	−119.51	−110.91
11	−118.48	−139.99	−133.84	−150.77	−145.99	−142.17	−110.66	−134.65	−150.39	−129.23	−159.95	−142.94	−120.18
12	−105.71	−104.13	−92.70	−97.49	−102.97	−123.80	−77.55	−92.01	−107.15	−101.17	−103.01	−106.00	−102.62
13	−120.23	−113.93	−104.08	−111.83	−114.14	−138.90	−90.10	−107.68	−119.68	−119.93	−113.92	−115.29	−109.72
14	−115.97	−116.58	−108.38	−111.39	−118.66	−138.22	−90.15	−112.18	−135.19	−114.99	−119.08	−123.39	−109.29
15	−154.64	−153.59	−143.40	−152.91	−147.30	−185.69	−120.53	−149.49	−162.06	−155.65	−169.29	−155.60	−148.29
16	−65.39	−78.88	−69.92	−90.53	−66.07	−83.24	−52.38	−61.49	−76.15	−76.08	−81.25	−72.75	−80.87
17	−79.36	−85.66	−75.61	−91.56	−81.05	−92.70	−60.22	−75.95	−91.47	−79.43	−98.19	−82.84	−86.38
18	−112.40	−105.11	−108.50	−132.26	−108.90	−113.23	−76.97	−98.29	−110.76	−98.19	−118.44	−99.45	−108.10
19	−72.31	−68.09	−73.83	−87.89	−73.29	−94.22	−53.90	−67.46	−80.10	−83.46	−74.85	−66.48	−61.55
20	−75.31	−75.67	−79.35	−90.25	−84.93	−100.31	−61.62	−74.38	−88.95	−77.73	−75.50	−78.49	−70.52
21	−91.41	−91.74	−105.27	−115.93	−105.56	−120.80	−74.26	−87.73	−106.98	−102.54	−97.56	−98.39	−101.47

*Vina*													
1	−7.30	−7.00	−7.80	−6.40	−7.60	−7.50	−7.20	−7.00	−7.90	−7.60	−7.40	−8.00	−8.50
2	−7.70	−7.40	−7.80	−6.90	−7.50	−7.50	−7.40	−7.30	−8.50	−7.50	−8.00	−8.60	−9.10
3	−6.00	−5.50	−5.70	−5.60	−5.60	−5.90	−4.90	−5.70	−5.90	−6.40	−6.90	−6.50	−5.00
4	−7.90	−7.60	−8.80	−6.50	−8.00	−7.50	−6.90	−8.10	−8.60	−7.60	−7.60	−8.70	−8.80
5	−7.60	−7.40	−8.40	−6.40	−8.10	−7.80	−7.10	−7.60	−8.00	−7.60	−6.60	−7.80	−8.80
6	−7.60	−7.50	−8.70	−7.20	−8.90	−8.50	−7.90	−8.20	−8.50	−8.50	−6.90	−8.30	−9.60
7	−6.60	−7.20	−6.90	−6.30	−6.70	−6.10	−5.80	−6.00	−7.10	−6.90	−7.80	−7.30	−6.70
8	−7.10	−7.70	−7.40	−6.30	−7.00	−7.50	−6.50	−6.90	−8.70	−7.50	−7.00	−8.50	−9.30
9	−6.90	−7.60	−7.90	−6.20	−7.30	−6.70	−6.70	−6.50	−8.10	−7.50	−7.10	−7.50	−7.70
10	−7.20	−6.90	−7.70	−6.90	−7.30	−7.30	−6.10	−7.10	−8.40	−7.80	−7.00	−7.70	−8.60
11	−5.00	−5.20	−5.70	−6.70	−7.10	−5.70	−4.40	−7.20	−7.90	−6.50	−6.90	−5.80	−6.20
12	−7.30	−7.70	−7.90	−6.30	−7.30	−6.50	−6.50	−6.60	−7.50	−7.50	−8.10	−7.20	−7.70
13	−7.50	−7.60	−7.90	−6.40	−7.80	−7.90	−6.30	−8.00	−8.10	−8.40	−6.80	−7.60	−8.70
14	−7.10	−7.10	−7.90	−7.40	−7.50	−7.60	−6.80	−7.30	−8.30	−7.60	−6.80	−7.90	−8.90
15	−7.20	−7.80	−8.60	−7.50	−8.00	−8.50	−6.80	−8.10	−8.20	−7.60	−9.50	−7.90	−7.70
16	−5.50	−6.00	−5.70	−5.70	−6.80	−6.70	−5.60	−6.80	−6.80	−6.20	−6.60	−6.80	−6.60
17	−6.30	−7.20	−7.10	−5.80	−6.80	−7.10	−5.20	−7.10	−8.00	−7.00	−6.30	−7.30	−7.60
18	−5.70	−5.50	−5.10	−5.30	−6.20	−6.70	−5.50	−5.90	−6.40	−6.20	−6.10	−6.30	−6.20
19	−5.80	−5.60	−5.10	−4.90	−6.10	−6.90	−5.50	−5.90	−6.30	−6.20	−6.10	−5.80	−6.20
20	−7.00	−7.30	−7.80	−6.80	−6.90	−7.50	−6.90	−7.60	−7.80	−7.60	−9.10	−7.50	−8.40
21	−6.90	−5.90	−6.30	−6.00	−6.80	−7.30	−6.50	−6.70	−7.40	−6.90	−7.20	−7.40	−6.60

*AutoDock-QC*													
1	−3.67	−7.64	−7.56	−5.58	−5.13	−7.08	−5.40	−4.64	−4.65	−4.68	−5.00	−4.18	−5.99
2	−3.65	−7.36	−7.43	−5.27	−5.70	−7.42	−5.57	−4.79	−4.65	−4.54	−4.78	−4.77	−6.46
3	−4.12	−2.96	−2.81	−3.02	−4.31	−3.62	−3.55	−3.94	−3.54	−3.65	−6.04	−3.78	−3.63
4	−4.29	−7.72	−7.53	−5.19	−5.70	−7.09	−5.93	−4.70	−4.67	−5.05	−5.38	−5.20	−5.81
5	−3.93	−3.91	−3.74	−3.46	−4.23	−3.70	−3.66	−3.84	−3.61	−4.05	−3.17	−4.78	−4.41
6	−4.14	−4.30	−4.23	−4.18	−5.60	−4.17	−3.84	−4.54	−3.77	−4.85	−2.09	−5.56	−5.44
7	−7.13	−6.24	−5.95	−4.93	−7.01	−6.02	−4.76	−6.20	−5.86	−6.43	−7.22	−6.13	−6.11
8	−3.27	−5.58	−5.76	−4.25	−4.84	−5.36	−5.34	−3.80	−3.45	−4.65	−5.89	−4.98	−5.90
9	−3.34	−6.34	−5.09	−4.24	−5.01	−5.15	−4.99	−3.91	−3.55	−4.78	−4.84	−4.13	−4.88
10	−3.04	−7.12	−6.41	−4.58	−4.82	−5.79	−4.87	−4.69	−4.06	−4.25	−5.27	−5.00	−4.84
11	−5.04	−4.10	−3.15	−3.85	−4.56	−4.16	−3.79	−4.35	−3.30	−4.06	−4.62	−5.14	−4.86
12	−2.93	−6.40	−6.44	−5.13	−5.48	−6.28	−5.47	−4.56	−4.79	−3.47	−4.70	−3.83	−5.76
13	−3.07	−6.79	−6.42	−5.14	−5.74	−5.50	−5.50	−4.41	−4.58	−4.14	−4.34	−4.33	−5.98
14	−3.19	−7.40	−6.67	−5.30	−5.45	−6.01	−5.37	−4.43	−3.67	−4.34	−5.02	−4.59	−6.51
15	−4.36	−4.21	−2.29	−2.91	−4.15	−4.28	−0.94	−1.59	−2.86	−2.58	−4.26	−3.93	−3.11
16	−3.79	−2.98	−2.84	−3.04	−3.75	−3.72	−2.97	−3.54	−2.98	−3.62	−3.92	−3.62	−3.90
17	−3.04	−3.38	−2.92	−2.44	−3.44	−3.29	−2.72	−3.18	−2.64	−3.67	−3.97	−3.97	−4.20
18	−3.98	−4.09	−3.21	−3.35	−3.81	−3.88	−3.40	−4.01	−3.31	−4.63	−4.01	−4.07	−4.67
19	−5.27	−2.87	−2.54	−2.70	−3.51	−2.95	−3.41	−3.30	−2.50	−2.72	−2.62	−2.96	−2.96
20	−2.12	−1.04	−1.26	−1.57	−2.33	−1.53	−2.43	−2.14	−2.67	−1.95	−2.01	−3.10	−2.43
21	−4.24	−1.64	−2.01	−2.32	−1.91	−1.86	−3.17	−3.13	−2.71	−2.32	−2.51	−3.22	−2.76

*AutoDock*													
1	−2.13	−5.32	−5.94	−4.59	−4.13	−4.99	−4.33	−3.91	−3.66	−2.46	−2.81	−2.64	−4.41
2	−3.99	−8.02	−7.99	−5.90	−6.81	−6.55	−5.06	−5.08	−5.19	−5.13	−5.72	−6.34	−7.17
3	−9.22	−2.68	−3.98	−3.67	−3.65	−3.65	−4.83	−4.70	−5.19	−6.23	−8.47	−5.08	−4.16
4	−2.03	−5.30	−5.41	−4.24	−4.26	−5.18	−4.25	−3.68	−3.66	−2.89	−2.62	−2.89	−4.05
5	−4.45	−4.22	−4.49	−3.85	−5.07	−4.33	−4.37	−4.58	−4.48	−5.04	−3.59	−5.44	−4.98
6	−4.20	−3.58	−3.69	−3.82	−4.55	−3.83	−3.63	−4.27	−3.21	−3.96	−0.70	−4.64	−4.33
7	−7.79	−1.14	−2.48	−2.05	−2.97	−0.53	−5.76	−4.36	−4.99	−7.07	−6.86	−5.61	−3.31
8	−3.99	−6.18	−7.49	−5.30	−6.32	−6.18	−5.65	−4.92	−4.08	−5.22	−6.41	−6.31	−7.84
9	−3.98	−7.81	−7.28	−5.25	−6.77	−5.64	−5.93	−4.11	−5.07	−5.12	−5.36	−5.42	−6.13
10	−2.32	−5.97	−5.77	−4.27	−3.41	−4.39	−3.69	−3.40	−2.79	−2.42	−2.95	−3.24	−4.17
11	−6.67	−9.90	−9.92	−7.88	−8.20	−9.19	−8.43	−7.47	−5.89	−7.95	−7.55	−9.80	−9.35
12	−3.27	−6.35	−6.30	−4.78	−5.76	−5.77	−4.89	−3.76	−5.33	−3.90	−4.85	−4.05	−6.06
13	−4.57	−7.87	−7.24	−6.00	−7.06	−6.02	−5.59	−6.06	−5.92	−6.06	−6.75	−5.89	−7.19
14	−3.20	−6.78	−6.39	−5.23	−5.35	−5.30	−4.51	−4.08	−3.68	−3.74	−4.48	−4.20	−6.16
15	−9.45	−2.73	−2.45	−3.20	−3.14	−2.25	−4.05	−5.84	−3.28	−7.44	−8.14	−5.60	−5.35
16	−5.75	−5.50	−4.59	−4.98	−5.91	−5.99	−4.29	−5.67	−4.88	−6.61	−6.21	−6.42	−6.77
17	−2.92	−3.50	−3.00	−2.97	−3.50	−3.06	−2.95	−2.86	−2.78	−4.04	−3.44	−3.88	−4.36
18	−5.27	−5.44	−4.03	−4.29	−5.01	−5.38	−4.17	−5.08	−4.14	−6.17	−5.48	−5.88	−6.03
19	−5.17	−5.42	−4.31	−4.52	−5.30	−5.55	−4.21	−5.42	−4.66	−5.83	−5.21	−5.46	−6.27
20	−8.96	−3.53	−3.87	−5.28	−4.15	−4.62	−5.39	−5.56	−6.39	−5.48	−6.94	−6.46	−5.56
21	−5.99	−4.99	−4.82	−4.71	−5.47	−6.25	−4.79	−5.88	−5.04	−6.30	−5.69	−6.21	−7.05

**Table 3:** The number of hydrogen bonds (HBs) in the interactions of the ligands **7**, **11**, and **15** with the receptors.

Receptor	Ligand **7**	Ligand **11**	Ligand **15**
CA-II	6	0	5
Cat B	8	1	3
DNA-1	5	2	7
DNA-2	2	2	5
Gyrase	3	1	6
HDAC7	3	0	3
HIS	4	1	4
Kinase	5	0	4
rHA	4	2	2
RNR	6	1	7
Top II	9	4	7
TrxR	6	4	3
TS	7	4	7

**Table 4:** The molecular properties of the modelled ligands.

	Energy	Zero energy	Thermal energy	Enthalpy	Gibbs free energy	CV	Entropy	HOMO	LUMO	Gap	Pol	Pol W	Hyp (esu)	Dipole
1	−226.22	−225.88	−225.88	−225.88	−225.91	13.89	65.46	−0.26	0.00	668.85	18.37	47.89	0.86	2.42
2	−304.87	−304.36	−304.35	−304.35	−304.39	26.38	85.47	−0.24	0.00	446.54	31.27	74.42	2.94	2.61
3	−603.38	−602.65	−602.64	−602.64	−602.68	34.79	98.26	−0.30	−0.09	563.27	42.55	92.52	1.84	3.55
4	−457.29	−456.58	−456.57	−456.57	−456.61	33.90	92.59	−0.22	−0.03	618.28	59.15	124.37	0.34	2.49
5	−490.54	−489.82	−489.81	−489.81	−489.85	33.65	96.73	−0.26	−0.02	622.64	106.83	109.44	1.69	3.58
6	−647.83	−646.77	−646.76	−646.76	−646.82	58.31	129.10	−0.23	−0.01	588.78	157.70	162.68	1.36	4.02
7	−1244.83	−1243.32	−1243.30	−1243.30	−1243.38	76.24	160.36	−0.30	−0.10	519.29	187.11	196.10	1.31	3.42
8	−952.69	−951.21	−951.20	−951.20	−951.27	73.73	150.85	−0.22	−0.04	473.61	265.57	273.88	2.71	3.21
9	−679.11	−678.19	−678.18	−678.18	−678.23	43.92	112.07	−0.26	−0.05	545.07	125.02	129.47	2.40	2.76
10	−836.40	−835.14	−835.12	−835.12	−835.19	68.58	141.09	−0.24	−0.05	503.87	175.52	182.12	2.47	3.59
11	−1141.26	−1139.59	−1139.57	−1139.57	−1139.65	84.04	166.49	−0.23	−0.06	447.56	284.93	296.55	4.19	2.35
12	−698.33	−697.32	−697.31	−697.31	−697.36	48.10	112.13	−0.24	−0.06	485.39	173.62	179.84	7.91	5.16
13	−886.90	−885.70	−885.68	−885.68	−885.74	58.91	129.78	−0.25	−0.10	411.44	196.81	205.34	11.21	3.21
14	−855.62	−854.28	−854.26	−854.26	−854.33	72.66	145.44	−0.23	−0.05	470.74	224.31	233.64	10.05	5.19
15	−1452.64	−1450.85	−1450.82	−1450.82	−1450.91	90.34	178.55	−0.27	−0.10	457.20	253.91	267.46	2.99	4.66
16	−495.41	−494.64	−494.63	−494.63	−494.67	35.89	94.59	−0.25	−0.06	502.88	133.18	136.67	0.48	3.24
17	−574.05	−573.11	−573.10	−573.10	−573.16	48.23	115.27	−0.24	−0.05	500.41	160.72	166.03	0.49	3.99
18	−872.57	−871.41	−871.39	−871.39	−871.45	54.72	120.56	−0.26	−0.10	428.47	177.26	184.39	1.61	2.67
19	−571.64	−570.77	−570.76	−570.76	−570.81	40.19	95.76	−0.24	−0.07	452.54	161.52	165.31	0.35	3.43
20	−650.29	−649.25	−649.24	−649.24	−649.29	52.75	116.19	−0.23	−0.06	500.97	191.88	197.84	3.40	2.30
21	−948.80	−947.54	−947.52	−947.52	−947.58	61.06	128.51	−0.26	−0.10	407.98	210.05	218.27	6.48	6.06

## Supplementary Materials

Supplementary Materials Figure S1: the binding site interaction of each of the three ligands **7** (grey), **11** (green), and **15** (yellow) (horizontally) with the receptors.
