# Supplementary material for: The Anticancer Activities of Some Nitrogen Donor Ligands Containing bis-Pyrazole, Bipyridine, and Phenanthroline Moiety Using Docking Methods
Source: Bioinorg Chem Appl. 2018 Jun 4;2018:5796287. doi: 10.1155/2018/5796287 (PMC6008838; doi:10.1155/2018/5796287)
Supplement: Supplementary Materials — Figure S1: the binding site interaction of each of the three ligands 7 (grey), 11 (green), and 15 (yellow) (horizontally) with the receptors. [file 5796287.f1.pdf]

**Supplementary Figures**

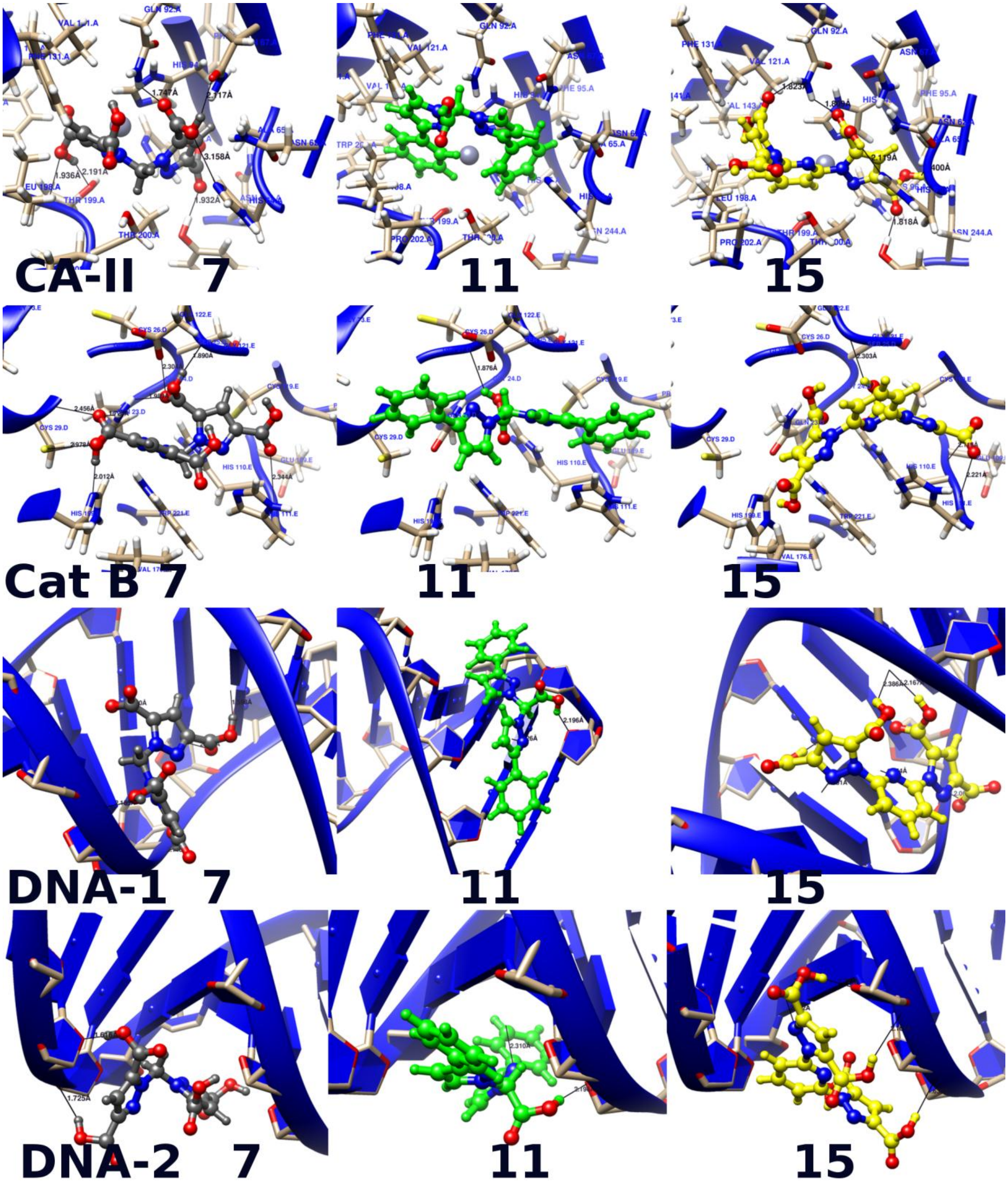

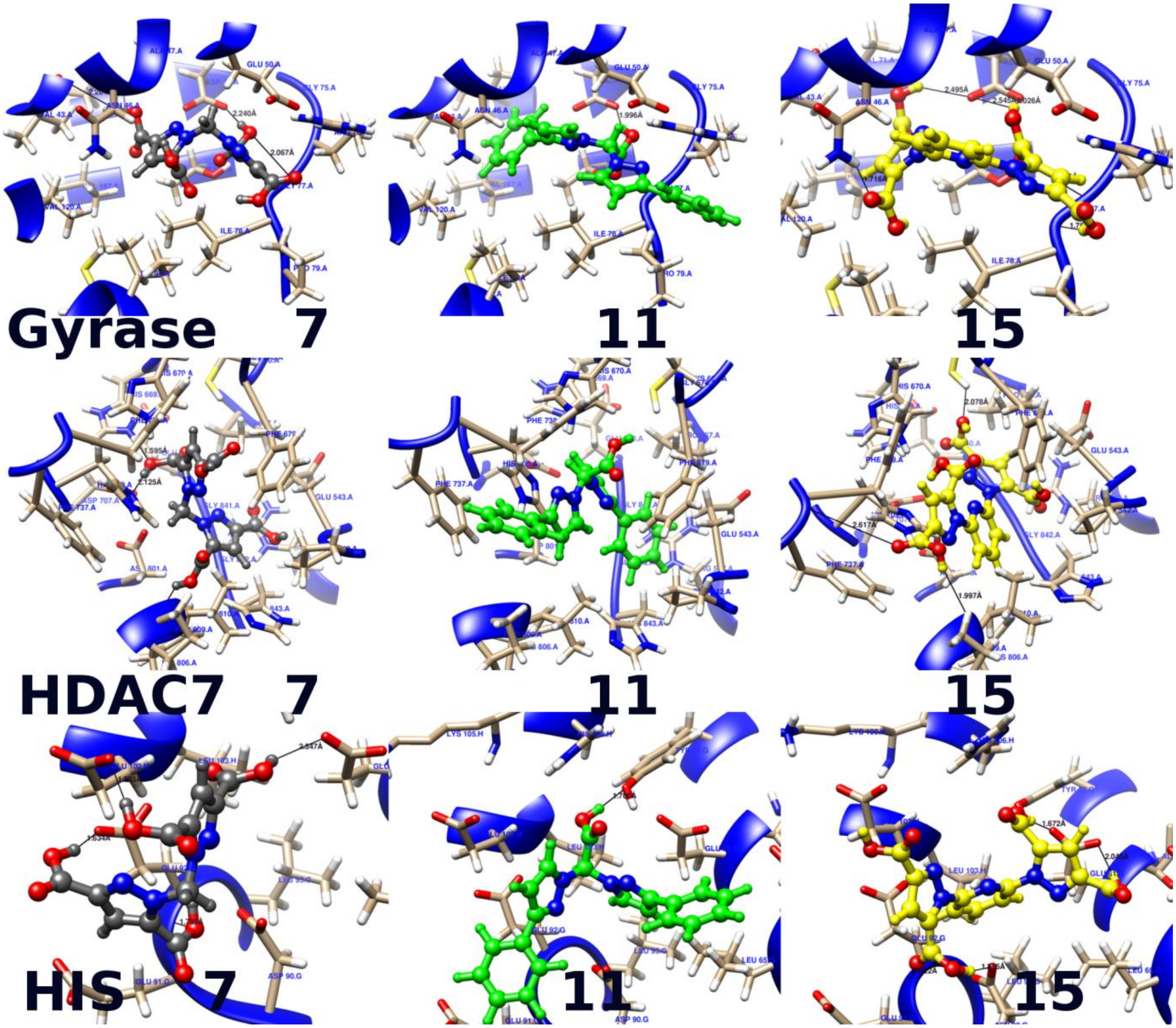
Gyrase
7
11
15
HDAC7
7
11
15
HIS
7
11
15

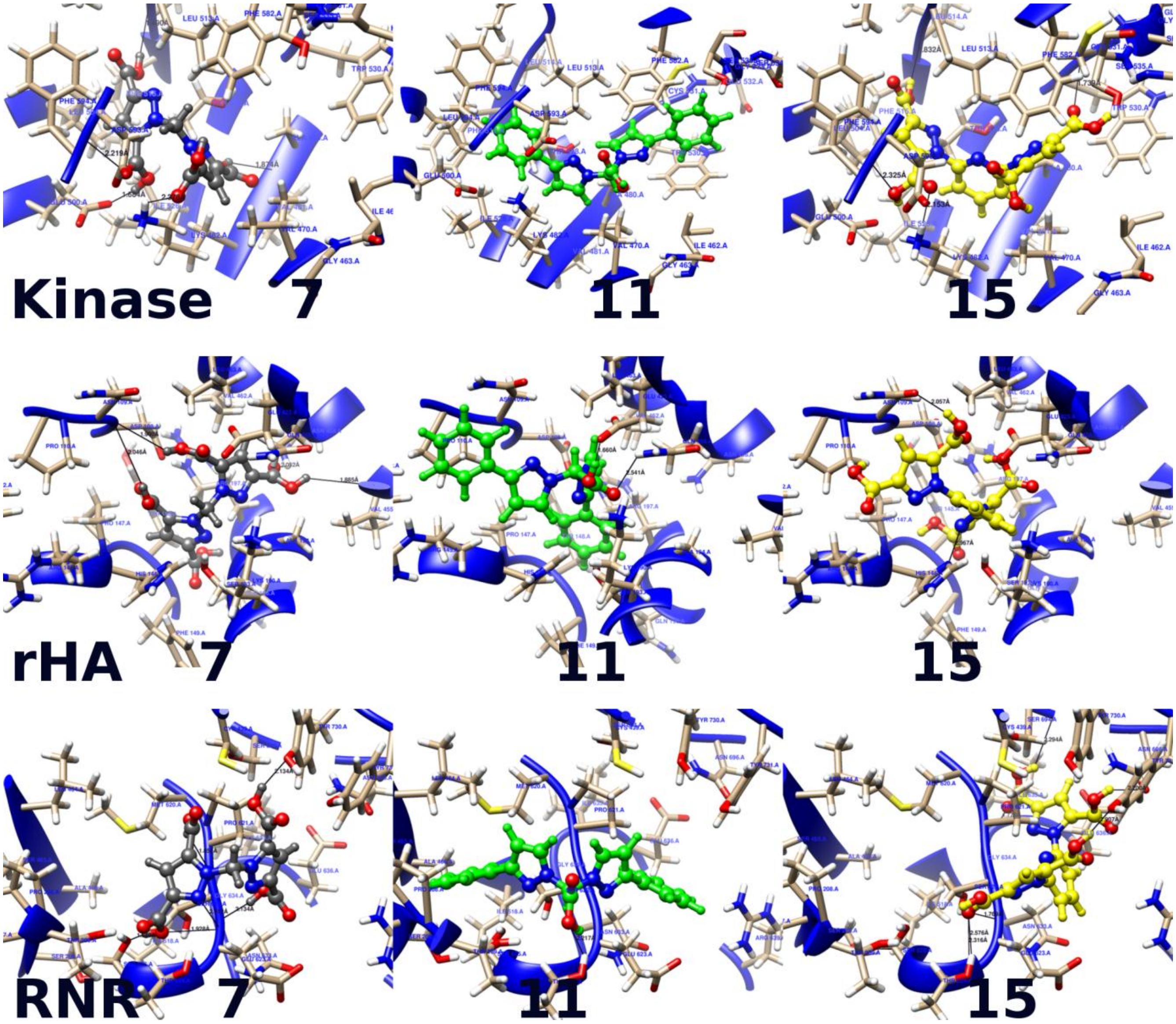
Kinase
7
11
15
rHA
7
11
15
RNR
7
11
15

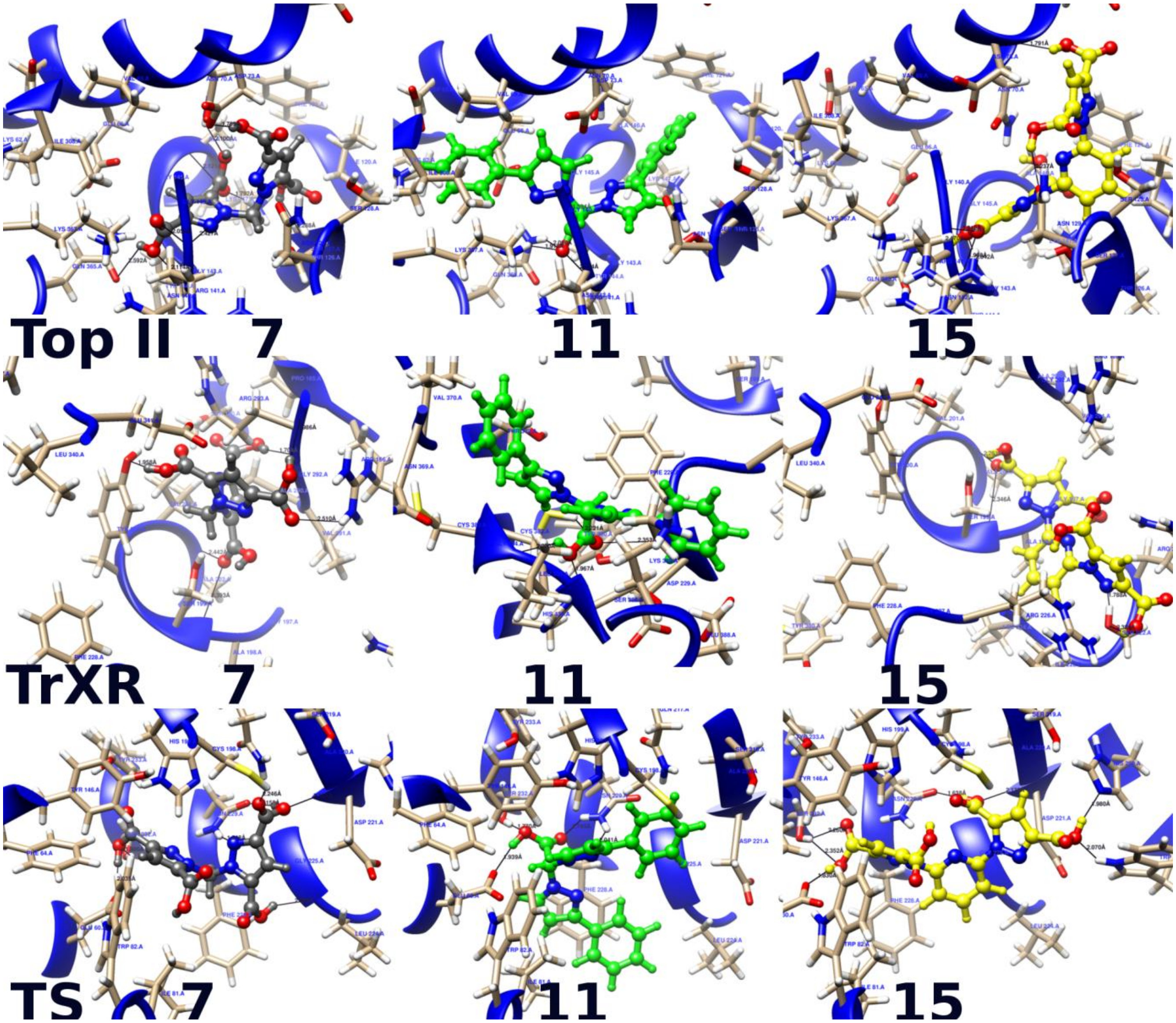


Figure S1: The binding site interaction of each of the three ligands 7 (grey), 11 (green) and 15 (yellow) (horizontally) with the receptors.
